# Biphasic role of 4-1BB in the regulation of mouse cytomegalovirus-specific CD8^+^ T cells

**DOI:** 10.1002/eji.200940256

**Published:** 2010-10

**Authors:** Ian R Humphreys, Seung-Woo Lee, Morgan Jones, Andrea Loewendorf, Emma Gostick, David A Price, Chris A Benedict, Carl F Ware, Michael Croft

**Affiliations:** 1 Division of Molecular Immunology, La Jolla Institute for Allergy and ImmunologyLa Jolla, CA, USA; 2 Department of Infection, Immunity and Biochemistry, School of Medicine, Cardiff UniversityCardiff, UK

**Keywords:** 4-1BB, CD8^+^ T cells, CMV, Memory

## Abstract

The initial requirement for the emergence of CMV-specific CD8^+^ T cells is poorly understood. Mice deficient in the cosignaling TNF superfamily member, 4-1BB, surprisingly developed exaggerated early CD8^+^ T-cell responses to mouse CMV (MCMV). CD8^+^ T cells directed against acute MCMV epitopes were enhanced, demonstrating that 4-1BB naturally antagonizes these primary populations. Paradoxically, 4-1BB-deficient mice displayed reduced accumulation of memory CD8^+^ T cells that expand during chronic/latent infection. Importantly, the canonical TNF-related ligand, 4-1BBL, promoted the accumulation of these memory CD8^+^ T cells, whereas suppression of acute CD8^+^ T cells was independent of 4-1BBL. These data highlight the dual nature of the 4-1BB/4-1BBL system in mediating both stimulatory and inhibitory cosignaling activities during the generation of anti-MCMV immunity.

## Introduction

CD8^+^ T cells are critical mediators of immunity to CMV infection. Virus reactivation in immune-compromised individuals correlates with defective CD8^+^ T-cell responses 1,2 and transfer of human CMV-specific CD8^+^ T cells limits viremia in immune-suppressed recipients 3,4. Interestingly, in healthy aging individuals, human CMV-specific CD8^+^ T cells expand to high numbers, comprising over 10% of the entire CD8^+^ repertoire 5,6. Although these T cells maintain their proliferative and cytolytic capacity 7, this enhanced frequency that dominates the global T-cell repertoire is associated with impaired T-cell responsiveness to heterologous antigens 8. As human CMV may impinge on immunity to other pathogens, understanding how such virus-specific CD8^+^ T-cell populations arise and are controlled may lead to new strategies to modulate these cells for the design of immune therapeutic and vaccination strategies. However, little is known regarding the immunological factors that determine the expansion and/or survival of distinct CMV-reactive T-cell subsets *in vivo*.

Due to the species-specific nature of HCMV replication, mouse CMV (MCMV) represents a useful system for modeling the pathogenesis and immunity of CMV infection *in vivo*. Similar to human CMV, MCMV persists in its natural host, following acute infection and establishes latency 9. CD8^+^ T-cell responses elicited by MCMV infection are well characterized and consist of cellular expansion and contraction of defined epitope-specific populations during acute infection, followed by an accumulation of distinct populations of “inflationary” CD8^+^ T cells thought to be representative of those observed in human CMV-infected individuals 10–13. Transfer of CD8^+^ T cells from immune donors also reduces MCMV replication in immune-suppressed recipients 14. Moreover, CD8^+^ T cells limit MCMV reactivation during latency 15.

4-1BB (CD137, TNFRSF9) is a member of the TNF receptor superfamily that is highly expressed by T cells following activation 16. Its known TNF-related ligand, 4-1BB ligand (4-1BBL, CD137L and TNFSF9), is expressed by activated APC 17,18. Following ligation *in vitro*, 4-1BB delivers a positive stimulatory signal to T cells promoting proliferation, survival and cytokine production. Evidence from some models of viral infection also suggests a critical role for the 4-1BB/4-1BBL pathway in modulating virus-specific CD8^+^ T-cell responses *in vivo*. Administration of an agonist antibody to 4-1BB was shown to enhance cytotoxicity and broaden the CD8^+^ T-cell repertoire during acute influenza infection 19. Additionally, endogenous 4-1BB/4-1BBL interactions have been shown to act late, after normal development of acute responses, to promote influenza-specific CD8^+^ T-cell memory formation, and also participate in either the maintenance and/or the reactivation of these persisting cells 20,21. More recently, 4-1BBL^−/−^ mice were found to generate impaired functional CD8^+^ T cells during latent mouse gammaherpesvirus-68 (MHV-68) infection although their numbers were not affected 22.

Despite the plethora of evidence suggesting that the 4-1BB/4-1BBL pathway acts as a positive regulator of CD8^+^ T-cell immunity, 4-1BB-deficient mice displayed hyper-responsiveness to immunization with some model protein antigens 23, and 4-1BB-deficient CD8^+^ T cells were found to expand to a greater extent to an antigen delivered *via* adenovirus, even when responding *in vivo* in a 4-1BB-sufficient environment 24. Furthermore, exogenous stimulation of 4-1BB by administering an agonist antibody at the time of LCMV infection was shown to inhibit rather than promote the generation of LCMV-specific CD8^+^ T cells 25. These contradictory observations highlight the need for a greater understanding of the role that 4-1BB plays in the regulation of anti-viral CD8^+^ T-cell responses *in vivo*. In this study, we assessed the impact that 4-1BB and 4-1BBL have on the initial generation of MCMV-specific CD8^+^ T cells.

## Results and discussion

### 4-1BB limits CD8^+^ T-cell accumulation during acute MCMV infection

WT C57BL/6 (B6) and 4-1BB-deficient mice were infected with MCMV and virus-specific CD8^+^ T-cell activity was measured, as identified by IFN-γ expression following *ex vivo* stimulation of splenocytes with H-2^b^-restricted peptides derived from either M38, M45, M57 or m139 MCMV proteins. During MCMV infection, the hierarchy of the CD8^+^ T-cell response shifts from an acute response predominated by cells recognizing M45, M57, and, to a lesser extent, m139-derived peptides, to a persistent response where m139 and M38-specific CD8^+^ T cells are immunodominant 26.

**Figure 1:**
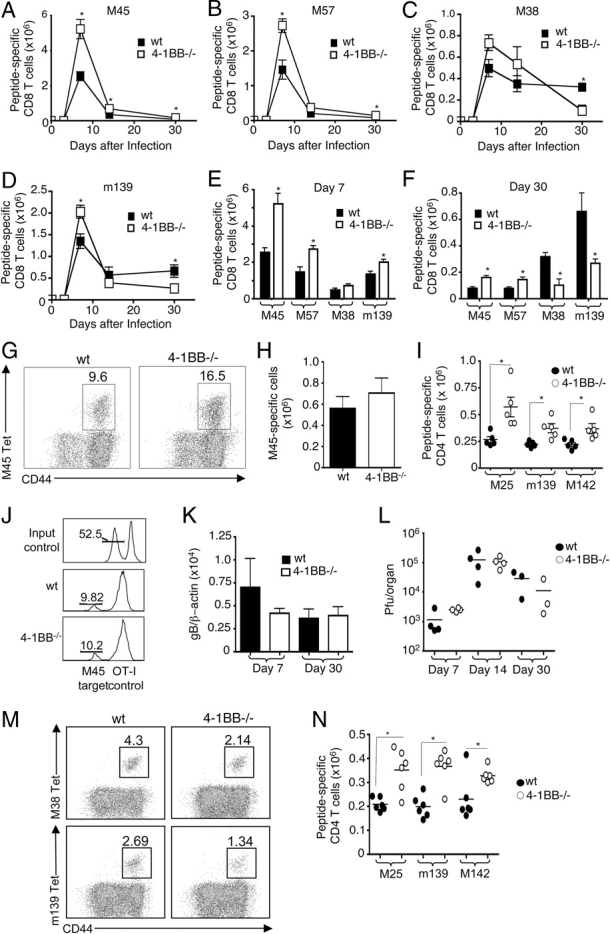
4-1BB^−^^/^^−^ mice have elevated early but reduced persistent MCMV-specific CD8 responses. WT C57BL/6 (▪) and 4-1BB-deficient (□) mice were infected with MCMV and on days 0, 7, 14 and 30 post-infection, CD8^+^ cells specific for M45 (A), M57 (B), M38 (C) and m139 (D) were quantified on the basis of intracellular IFN-γ production (E and F). Numbers of peptide-specific CD8^+^ cells 7 (E) and 30 (F) days post-infection. Results are expressed as numbers of peptide-specific CD8^+^ cells/spleen and are shown as mean±SEM of four mice/group, representing three independent experiments. (G) Representative plots of M45-specific tetramer-binding CD44^+^ CD8^+^ T cells from WT (left) and 4-1BB^−/−^ mice 7 days post-infection. Results represent eight mice from two experiments. (H) Splenic M45-specific CD8^+^ cell numbers 7 days after immunization with M45 peptide/CFA. Mean±SEM of four mice/group is shown. (I) Numbers of peptide specific CD4^+^ cells 7 days post-infection. Individual mice and mean values are shown, and data represent two independent experiments. (J) *In vivo* CTL assay as described in the *Materials and methods* section. Representative plots of loaded cells prior to transfer (top) and from MCMV-infected WT (middle) and 4-1BB^−/−^ (bottom) mice 7 days post-infection are shown, and represent four mice/group. (K) MCMV glycoprotein B content in genomic DNA from spleens of WT and 4-1BB^−/−^ mice 7 and 30 days post-infection was measured by qPCR and normalized to β-actin. Results are expressed as mean±SEM of three mice/group. (L) Infectious viral load in salivary glands was measured by plaque assay. Individual mice and mean values are shown. (M) Representative plots of M38- and m139-specific tetramer-binding CD8^+^ T cells from WT (left) and 4-1BB^−/−^ (right) mice 30 days post-infection. Results represent 12 mice from two independent experiments. (N) Numbers of peptide specific CD4^+^ T cells 30 days post-infection. Individual mice are shown and data represent two independent experiments. Significance is ^*^p<0.05, Student's *t*-test.

At day 7, the numbers of MCMV-specific CD8^+^ T cells responsive to M45 (Fig. 1A), M57 (Fig. 1B) and m139 (Fig. 1D) were surprisingly elevated in the spleens (Fig. 1E) and lungs (data not shown) of 4-1BB^−/−^ mice; this was particularly evident with M45 and M57 populations, and was also observed when tetramers loaded with peptides of M45 (Fig. 1G) or m139 (data not shown) were used to identify virus-specific CD8^+^CD44^+^ T cells. These observations directly correlate with our earlier finding that 4-1BB-deficient CD8^+^ T cells expanded to a greater extent to an antigen expressed in adenovirus 24. Very low numbers of M38-specific CD8^+^ T cells were detected in both groups of mice (Fig. 1C and E). Interestingly, and in contrast to the infection data, increased accumulation of M45-specific CD8^+^ T cells in 4-1BB^−/−^ mice was not observed following peptide immunization (Fig. 1H), suggesting that the inhibitory function of 4-1BB is only apparent under particular conditions and that MCMV infection promotes this activity.

Increased CD8^+^ T-cell responses in MCMV-infected 4-1BB^−/−^ mice were not accompanied by increased numbers of NK cells at 3 and 7 days post-infection (data not shown). On the contrary, virus-specific splenic CD4^+^ T cells were elevated in 4-1BB^−/−^ mice 7 days post-infection (Fig. 1I). Importantly, however, acute MCMV-specific CD8^+^ T-cell responses are not dependent on CD4^+^ T cells 27,28, implying that 4-1BB directly suppresses the priming of acute MCMV-specific CD8^+^ T-cell populations, rather than acting indirectly by modulating CD4^+^ T-cell activity. The enhanced accumulation of MCMV-reactive CD8^+^ T cells might have been a consequence of modulating the viral load. However, as described above, early splenic NK cell numbers were not altered in the absence of 4-1BB, implying that early antiviral protection should have been comparable in WT and 4-1BB^−/−^ mice. Also, lysis of M45 peptide-loaded target cells was equally efficient following transfer into MCMV-infected WT and 4-1BB^−/−^ mice (Fig. 1J) and, in accordance, viral burden in the spleen (Fig. 1K) and salivary gland (Fig. 1L) was not significantly different between WT and 4-1BB^−/−^ mice at day 7 post-infection. These observations suggest that elevated numbers of MCMV-reactive CD8^+^ T cells were not a consequence of altered exposure to viral antigens, and that a ∼2-fold increase in virus-specific CD8^+^ T-cell numbers does not reduce MCMV burden during acute infection of 4-1BB^−/−^ mice.

### 4-1BB promotes MCMV-specific CD8^+^ T-cell memory responses

Next, we studied CD8^+^ T-cell responses at later times after infection. The populations of acute M45- and M57-specific CD8^+^ T cells contract rapidly after 7–8 days 26, and a deficiency in 4-1BB did not overtly abrogate this process. However, a small increase in the numbers of these stable M45- and M57-specific memory populations was observed 30 days post-infection in the spleens (Fig. 1A, B and F) and lungs (data not shown) of 4-1BB^−/−^ mice. Strikingly, and in contrast to the acute phase of infection, the accumulation of “inflationary” IFN-γ^+^ M38- and m139-specific CD8^+^ T cells during the early phase of latent/chronic infection (day 30) was reduced in the spleens of 4-1BB^−/−^ mice compared with WT controls (Fig. 1C, D and F). Similarly, M38-specific CD8^+^ T-cell numbers were reduced in the lungs of 4-1BB^−/−^ mice at this time (data not shown). Moreover, reduced frequencies of tetramer binding inflationary CD8^+^ T cells were observed in 4-1BB^−/−^ mice (Fig. 1M), suggesting that 4-1BB regulated the accumulation rather than function (IFN-γ production) of these memory cells. Virus load at 30 days was comparable in spleens (Fig. 1K) and salivary glands (Fig. 1L) of WT and 4-1BB^−/−^ mice, suggesting that impaired T-cell inflation in 4-1BB^−/−^ mice was again not a consequence of reduced antigen abundance. CD4^+^ T cells contribute to the size of these late CD8^+^ T-cell populations 27,28. Surprisingly, however, accumulation of virus-specific CD4^+^ T cells was actually enhanced in 4-1BB^−/−^ mice (Fig. 1N), suggesting that impairment of memory CD8^+^ T-cell responses in these mice was not a consequence of reduced CD4^+^ T-cell help. Collectively, these data show that 4-1BB has a biphasic action by limiting CD8^+^ T-cell priming during acute MCMV infection yet promoting inflationary CD8^+^ T-cell accumulation at later stages of infection.

### 4-1BBL does not contribute to 4-1BB-mediated suppression of acute CD8^+^ T-cell responses

**Figure 2:**
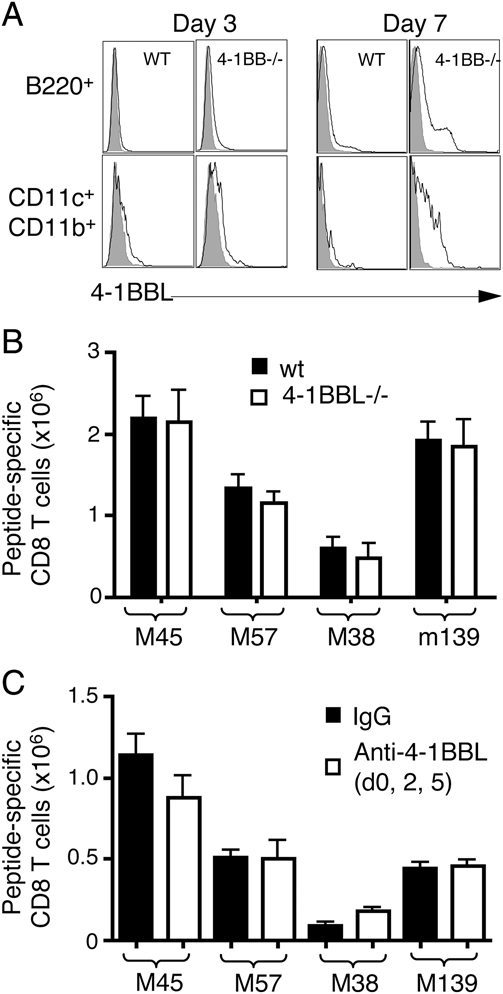
4-1BB-mediated suppression of early anti-viral CD8^+^ T cells is independent of 4-1BBL. (A) WT and 4-1BB^−/−^ mice were infected with MCMV and expression of 4-1BBL on B220^+^ and CD11b^+^CD11c^+^ cells was measured by flow cytometry after 3 (left panels) and 7 (right panels) days. Closed line, isotype; open line, α4-1BBL. (B) WT (▪) and 4-1BBL^−/−^ (□) mice were infected with MCMV and numbers of virus-specific IFNγ^+^ CD8^+^ cells were enumerated on day 7. (C) WT mice were treated with IgG (▪) or α4-1BBL (□) on days 0, 2 and 5, and virus-specific IFNγ^+^ CD8^+^ cells were enumerated on day 7. All results shown are mean numbers±SEM of four mice/group and represent two to three independent experiments. ^*^p<0.05, Student's *t*-test.

We next investigated whether 4-1BBL played a role in these divergent responses revealed in the absence of 4-1BB. Analyzing B6 and 4-1BB^−/−^ mice (in which surface expression of 4-1BBL is stabilized 29), we observed that 4-1BBL was expressed by B220^+^ and CD11b^+^CD11c^+^ cells during acute infection, although it was more abundant at late times (day 7) rather than at early times (Fig. 2A). Analysis of 4-1BB-deficient mice at day 7, but not at day 3, showed a much higher level of 4-1BBL expression than in WT mice, implying that productive 4-1BB/4-1BBL interactions (that can result in cleavage of surface 4-1BBL) were occurring late but not early during initial infection. Correlating with this, when acute CD8^+^ T-cell responses were examined in 4-1BBL-deficient mice, we found no defect in the generation of any of the MCMV-reactive populations compared with WT controls (Fig. 2B). Furthermore, this result was replicated in WT mice treated with a blocking antibody to 4-1BBL given during the first week of infection (Fig. 2C). These results show that the suppressive activity of 4-1BB on acute CD8^+^ T-cell responses occurs independently of 4-1BBL.

### 4-1BBL promotes MCMV-specific memory CD8^+^ T-cell accumulation

**Figure 3:**
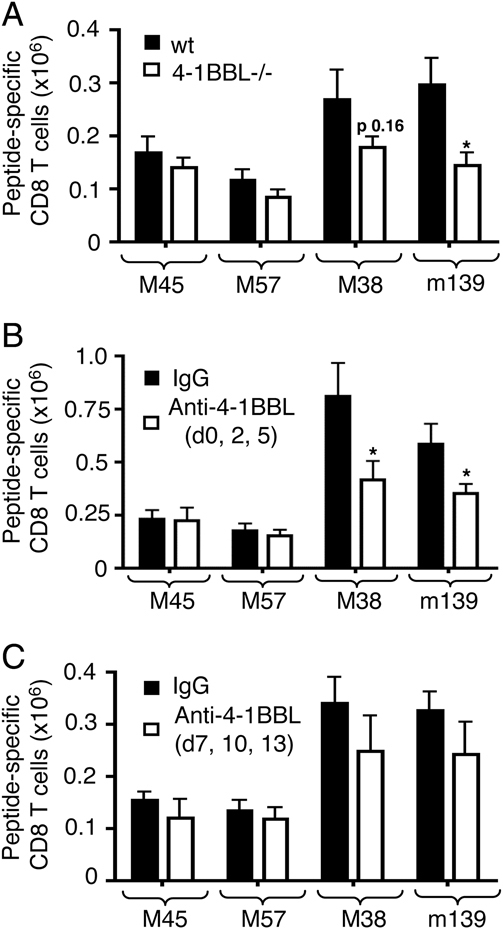
4-1BB/4-1BBL interactions during acute infection promote CD8 persistence. (A) WT (▪) and 4-1BBL^−/−^ (□) mice were infected with MCMV and numbers of virus-specific CD8^+^ cells were enumerated functionally 30 days later. (B and C) MCMV-infected WT mice were treated with IgG (▪) or α4-1BBL (□) antibody on days 0, 2 and 5 (B) or 7, 10 and 13 (C) and MCMV-specific CD8^+^ cells were enumerated functionally after 30 (B) or 28 (C) days. All results shown are mean numbers±SEM of four to six mice/group and representative of two independent experiments. ^*^p<0.05, Student's *t*-test.

Given that 4-1BBL promotes the generation of some anti-viral memory CD8^+^ T-cell populations in mice, and 4-1BBL binding to 4-1BB induces the expansion of human HCMV-specific CD8^+^ memory T cells *in vitro* 30, we investigated whether 4-1BBL might control the later accumulation of inflationary CD8^+^ T cells. Thirty days after infection, 4-1BBL^−/−^ mice displayed reduced accumulation of these persistent CD8^+^ T-cell populations (Fig. 3A), similar to the defect seen in 4-1BB^−/−^ mice (Fig. 1F). Furthermore, we found that treatment of WT mice with a blocking α4-1BBL antibody given on days 0–5 (Fig. 3B), but not days 7–13 (Fig. 3C), post-infection, also reduced the MCMV-specific CD8^+^ T-cell responses measured at 1 month, suggesting that the requirement for and activity of 4-1BBL likely occurred just before or at the peak of the effector T-cell response in the first week of infection, correlating with the expression data above. As seen in 4-1BB^−/−^ mice, impaired T-cell inflation following early 4-1BBL blockade was not associated with reduced MCMV genome load in the spleen (data not shown). Some variability in T-cell responses was seen between experimental groups, such that statistical significance was not always achieved. However, combining the 4-1BBL knockout and 4-1BBL-blocking studies together essentially replicated the defective accumulation of both M38 and m139-reactive CD8^+^ T cells that were seen in the absence of 4-1BB. Thus, acute MCMV-specific CD8^+^ T-cell responses are negatively regulated by 4-1BB, but independent of 4-1BBL, whereas late CD8^+^ T-cell responses are positively regulated by 4-1BB and dependent on 4-1BBL.

As the inhibitory action of 4-1BB on acute CD8^+^ T-cell responses was proven to be independent of its interaction with 4-1BBL, this leads to several possible conclusions. First, 4-1BB might function in an autonomous manner, irrespective of engagement by a ligand, perhaps as part of another receptor complex. Alternatively, and the explanation we favor, is that the suppressive action of 4-1BB depends on the presence of another as yet unidentified binding partner. We had previously hypothesized the existence of such a partner based on the unusual hyper-reactivity reported in 4-1BB^−/−^ mice 23,24 that had never been observed in publications with 4-1BBL^−/−^ mice. The direct comparison here between the knockout strains represents the first instance where divergent activities have been seen in the same model. Briefly, 4-1BB binds several extra-cellular matrix proteins, including laminin 31. However, given the ubiquitous expression of ECM proteins, it is unlikely that these will account for the selective kinetics of 4-1BB suppression. Any novel interaction with 4-1BB may operate in several ways. It may suppress CD8^+^ T cells by inducing a negative signal through 4-1BB expressed on the T cell. Alternatively, it might induce an inhibitory signal in the T cell through the alternate ligand, following either cis- or trans-interactions with 4-1BB expressed on a neighboring T cell or APC, respectively. Another possibility is that 4-1BB stimulates an APC or regulatory population, through 4-1BB or its alternate partner, leading to expression of a suppressive molecule. Importantly, in terms of viral immunity, 4-1BB-mediated inhibition of CD8^+^ T-cell priming was not observed following M45 peptide immunization, or in response to vaccinia virus (data not shown), highlighting the activity of MCMV in eliciting this inhibitory function. It is conceivable that MCMV expresses a 4-1BB-binding protein that preferentially induces negative signaling. More likely, MCMV may induce the expression of a host factor(s) that upregulates a 4-1BB binding ligand. Irrespective, identifying any novel ligands for 4-1BB, and understanding the mechanisms by which they might promote 4-1BB-mediated suppression of T cells, will be crucial for our understanding of how 4-1BB regulates anti-MCMV immune responses.

Our further conclusions show that MCMV-specific CD8^+^ T-cell memory formation is promoted by 4-1BB/4-1BBL costimulatory events that occur during acute infection when, paradoxically, 4-1BB concurrently inhibits primary CD8^+^ T-cell populations in a 4-1BBL-independent manner. It is possible, however, that these events are temporally distinct. One idea is that M45- and M57-specific populations expand quickly because the peptides are readily available, but that M38- and m139-specific populations exhibit delayed expansion kinetics of development as the peptides are either not presented immediately or are less abundant. The suppressive activity of 4-1BB might dominate early, resulting in diminished responses to M45 and M57 responses, because 4-1BBL is not expressed at high levels during the very early phase of infection. On the contrary, at later times during the acute response, perhaps coinciding with maximal M38 and m139 peptides presentation enhanced levels of 4-1BBL might result, switching the activity of 4-1BB from being anti-inflammatory to pro-inflammatory and hence aiding the generation of CD8^+^ T cells to these epitopes.

## Concluding remarks

Our data add to the significance of the literature, showing that human CD8^+^ T cells recognizing peptides derived from influenza, EBV 32 and HIV 33 can be promoted by providing 4-1BBL to engage 4-1BB, and similarly that HCMV-specific effector memory CD8^+^ T cells can respond to 4-1BB signals 30. However, the observation that 4-1BB exerts differential effects on anti-viral CD8^+^ T-cell responses at varying stages of infection, and that 4-1BBL is dispensable for early suppression of CD8^+^ cells, underlines the complexity of the 4-1BB/4-1BBL pathway and highlights the need for greater understanding before manipulating these molecules to promote anti-viral immunity in the clinic.

## Materials and methods

### Mice

C57BL/6 and C57BL/6^Pep3b/BoyJ^ (CD45.1^+^) mice were purchased from The Jackson Laboratory. 4-1BB^−/−^ and 4-1BBL^−/−^ mice were originally provided by Byoung Kwon and Amgen, respectively. All experiments were conducted following the guidelines of the La Jolla Institute for Allergy and Immunology's Institutional Animal Care and Use Committee.

### Virus, mouse infections and treatments

MCMV Smith strain (ATCC) was prepared, isolated and titred from salivary glands as described previously 27. Mice were infected i.p. with 5×10^4^ pfu MCMV. In some experiments, mice were also injected with 250 μg rat IgG (Chemicon) or anti-4-1BBL (clone 19h3, a kind gift from Robert Mittler) on days stated in the figure legends. In other experiments, mice were immunized with 25 μg peptide in 100 μL CFA s.c. tail base.

### Flow cytometry

Peptide-specific IFN-γ-expressing CD8^+^ T cells were measured as described previously 29. To examine tetramer-binding CD8^+^ T cells, 1×10^6^ splenocytes were incubated with Fc block and then stained with M45/H-2D^b^, M38/H-2K^b^ or m139/H-2K^b^ tetramers. Cells were then stained with αCD3-PerCP-Cy5.5 (BD Pharmingen) and αCD8-APC-H7 (BD Pharmingen).

To identify MCMV-specific CD4^+^ T cells, splenocytes were incubated for 5 h at 37°C with 2 μg/mL brefeldin A (Sigma-Aldrich) and 3 μg/mL MHC class II restricted MCMV-derived peptides (Genscript) derived from the M25, m139 and M142 proteins 34. Cells were then incubated with Fc block and surface stained with αCD4-Pacific Blue (BD Pharmingen) prior to permeabilization and intracellular staining with αIFN-γ-FITC. All data were acquired on an LSR II flow cytometer (BD Bioscience) and analyzed with FlowJo software.

### *In vivo* CTL assay

CD45.1^+^ splenocytes were loaded with M45 or OT-I (SINFEKL) peptide (5 μg/mL) for 60 min and labeled with CFSE^lo^ (0.5 μM) and CFSE^hi^ (5 μM), respectively. Five million CD45.1^+^ target cells (2.5×10^6^ each) were adoptively transferred to MCMV-infected WT and 4-1BB^−/−^ mice 7 days after infection. Spleen cells were isolated 3 h after transfer and CD45.1^+^CFSE^+^ target cells were quantified by flow cytometry.

### Viral genome detection

Genomic DNA was isolated from spleen and lung tissue using a DNAeasy tissue kit (Qiagen). MCMV glycoprotein B was then assayed by quantitative PCR using a Mini Opticon (Biorad Laboratories) and Platinum SYBR green mastermix reagent (Invitrogen). In total, 100 ng aliquots of DNA were used as templates for each reaction. The primer sequences are available upon request.
